# Supplementary material for: Decoding kinase-adverse event associations for small molecule kinase inhibitors
Source: Nat Commun. 2022 Jul 27;13:4349. doi: 10.1038/s41467-022-32033-5 (PMC9329312; doi:10.1038/s41467-022-32033-5)
Supplement: Supplementary file 1 — Supplementary Information [file 41467_2022_32033_MOESM1_ESM.pdf]

SUPPLEMENTARY INFORMATION

Decoding of Kinase-Adverse Event Associations for Small Molecule Kinase Inhibitors

Xiajing Gong[1], PhD; Meng Hu[1], PhD; Jinzhong Liu[1], PhD; Geoffrey Kim[2*], MD; James Xu[3*], MD; Amy McKee[4*], MD; Todd Palmby[2*], PhD; R. Angelo de Claro[1], MD; Liang Zhao[1**], PhD

1. Center for Drug Evaluation and Research, Food and Drug Administration, Silver Spring, MD
2. BeiGene, Cambridge, MA
3. Potomac Oncology and Hematology, Rockville, MD
4. Parexel, Washington, DC.

*Drs. Kim, Xu, McKee, and Palmby participated in this work while employed at the U.S. Food and Drug Administration.

****** Correspondence to: Liang Zhao, PhD Liang.Zhao@fda.hhs.gov

Evaluation of AE prediction performance using DeepHit and ANN

We applied a recently developed deep-learning based survival analysis method (i.e., DeepHit) to our data and compared performances between RSF and DeepHit. We also added to the performance evaluation a well-developed ML method, artificial neural network (ANN), which had been used for ML method demonstration for time-to-event analysis in our previous simulation work [1].

DeepHit

Unlike conventional survival models that rely on strong parametric assumption, DeepHit uses a deep neural network to learn the distribution of survival times directly and makes no assumptions about the underlying stochastic process and allows for the possibility that the relationship between covariates and risk(s) changes over time. Like parametric approaches, DeepHit can accommodate multiple competing risks [2].

An exemplary architecture of a DeepHit model dealing with two events of interest is shown in Supplementary Fig. 1. The DeepHit model consists of multiple fully connected subnetworks, comprising a shared sub-network and multiple cause-specific sub-networks. The shared sub-network inputs information from covariates ($\mathbf{X}$), and the cause-specific sub-network outputs information from the shared sub-network and covariates to the outcome information of the specific event. Specifically, to handle survival data, the DeepHit model takes in a covariate matrix $\mathbf{X}$, and outputs a vector $\mathbf{y}$ where each element $y(k, t)$ represents the probability of a certain event k (e.g., death or cured) occurring at time t. Covariate matrix $\mathbf{X}$ first enters one shared subnetwork, and then gets through cause-specific subnetworks for the competing risks considered. To make sure information in the original covariate matrix $\mathbf{X}$ is not lost, there is a residual connection between $\mathbf{X}$ and each of the cause-specific subnetworks. The output layer is a SoftMax layer, so the sum of $y(k, t)$ equals 1. More detailed information about the DeepHit model can be found in the literature [3] and at https://github.com/chl8856/DeepHit.

The DeepHit model was applied to our data for performance comparison. We customized the DeepHit code shared by the developer and conducted data analysis in a TensorFlow environment. For hyper-parameter finetuning, we adopted a random search strategy, consisting of 20 random search iterations. A random combination of the following hyperparameters is selected in each iteration: number of neurons and number of layers in shared and cause-specific subnetworks, activation function, and beta (i.e., a value of the ranking loss function that is adapted from the idea of concordance and represents number of correctly ordered pairs). After building a model for each of the 20 random combinations of the hyperparameters, the models are evaluated on the test dataset, and the hyperparameters corresponding to the model with the highest C-index on the testing set are selected. We built one DeepHit model for each adverse event with its own set of hyperparameters.

ANN

ANNs mimic brain networks to implement artificial intelligence learning. An ANN typically consists of three layers: input (containing nodes for the predictor variables), hidden (containing nodes for mapping input to output layers), and output (containing nodes for the prediction outcomes). The nodes between adjacent layers are fully connected. The strength of each connection is usually represented by an adjustable weight parameter. Each node in the hidden

and output layers can serve as a nonlinear activation function (e.g., logistic function), of the weighted linear combination of inputs from the previous layer. Application of the standard ANN to survival data is not straightforward and requires adaptation. In our study, we used the partial logistic regression approach ANN (PLANN) [4] based on a three-layer, feed-forward neural network among previously proposed ANN strategies for survival analysis [5, 6]. Briefly, to accommodate survival data in ANN, the input layer in this model includes both the predictor variables of interest and the time variable. A series of non-overlapped time intervals covering the duration of the study are predefined. For each subject, the values of his/her predictor variables are replicated for all time intervals. Uncensored cases are repeated until a time interval is reached in which the event is observed. During training, the output layer uses '0' for each time interval without event, and '1' for the time interval with event. Given a trained PLANN, the output can yield the approximated hazard function and survival function. In principle, PLANN model allows the joint modeling of time and the continuous and categorical covariates as input predictors without proportionality constraints.

Results

We evaluated the predictive performance of the ML-based methods using the same dataset described in Supplementary Table 2. For each AE, 80% of the dataset were bootstrap-sampled as the training dataset, and the remaining 20% of the dataset were used for model validation. Supplementary Table 2 shows the predictive performance as measured by C-index and 90% confidence interval. we investigated five more AEs to compare the prediction performances across different ML methods. The three ML methods showed generally similar performances. While no ML method stands out as a clear "winner" in terms of prediction performance, RSF seems to perform slightly better by providing more highest C-index scores among all the cases (i.e., 4 out of 10) compared to other two methods (i.e., 3 out of 10).

Caveat should be given to the use of DeepHit. As a method not originally developed for the purpose, it can be challenging to use DeepHit to determine the variable importance. To counter the challenge, Dynamic DeepHit, an extension of DeepHit, was developed to assess variable importance [2]. Briefly, to test the importance of a variable, two models are established by using the maximum and minimum values of the interested variables individually. The variable importance is represented by calculating the difference in cumulative incidence functions of the two models. However, we found that the variable importance algorithm for Dynamic DeepHit can only effectively identify important variables when these variables are linearly related to dependent variables. In summary, an efficient variable importance method for DeepHit has not been developed. The permutation approach could be another option for variable importance analysis, but further efforts are warranted.

Literature survey on associations between kinase targets and AEs

To investigate kinase-associated toxicities reported in clinical studies, a comprehensive literature survey has been conducted as follows:

1. We conducted a query of PubMed database with Boolean formula (((adverse event[OT]) OR (adverse effect[OT]) OR (adverse reaction[OT]) OR (toxicities[OT])) AND (kinase [OT]) AND 2013:2022 [dp]) and collected 88 papers as a result

2. Out of the 88 papers, 63 papers describing association between kinase and AEs in their abstracts were selected for further review.
3. In addition, 15 papers citing the 2013 review study [7] were also selected.
4. Among the 78 selected articles, all the mentioned KI-AE pairs were manually extracted.

We then examined the list of the kinase-associated toxicities following this literature survey effort against the model-identified KI-AE pairs shown in main text Figure 2a. The predicated KI-AE pairs found to be validated by the more recently published experimental evidence (post [7]) are highlighted yellow in Figure 2a; the corresponding studies that reported the KI-AE association are summarized in Supplementary Table 3.

Comparison of the predictions from ML models with AEs reported in FAERS

As additional validation of ML model prediction, we compared the predictions from ML models with AEs reported in the FDA Adverse Event Reporting System (FAERS), a post-marketing surveillance database. The comparison process is as follows:

We first conducted queries in the U.S. FAERS database for the safety profile regarding the two SMKIs neratinib and imatinib. The search parameters are listed In Supplementary Table 4. Based on the query results (provided in Supplementary Dataset 1.xlsx), we extracted the total number and percentage of the reported AEs.

To compare the AEs reported in FAERS with our predictions, we used the same data used to generate Figure 3 in the main text, specifically data from patients suffering from:

- breast cancer in two neratinib monotherapy studies (116 and 136 patients respectively),
- chronic myelogenous leukemia in an imatinib monotherapy study (349 patients).

We applied the trained ML model to predict patient-level AE probabilities for these studies and then calculated the averaged AE prediction across study population.

Of note, the ML predictive model in this study was based on randomized controlled clinical trial data while the FAERS data is derived from a spontaneous post-marketing reporting system. Given the different nature of the data, we did not directly compare their results by absolute percentages but conducted comparative analysis by calculating (i) hit rate of ML model predicted AEs with high probability (>= 10%) in a top 20 reported AEs in FAEARS (two columns from the rightmost in Supplementary Table 5), (ii) Spearman rank correlation coefficient (Spearman ρ) of the occurrence percentages between the ML model predicted AEs and the top 20 reported AEs in FAEARS. Spearman ρ is used as it is based on rank order of the data, and can accommodate a certain level of nonlinear correlation, which is considered more appropriate for the real-world situation.

Supplementary Table 5 shows results of whether an AE with ML model prediction of high probability (i.e., >=10%) appears in the top 20 reported AEs in FAEARS ("Yes" in 1st column). For neratinib study I (Supplementary Table 5.a), among the top 20 reported AEs in FAEARS, 13 AEs were predicted as AEs with high probability (>=10%), 4 AEs were not reported in the clinical studies. Though there are three mismatched AEs (i.e., dehydration, abdominal discomfort, and muscle spasms), they are at the relatively lower ranks in the FAERS top 20 list (i.e., 6.6%, 4.8%

and 4.2%). Similar results were observed for neratinib study II (Supplementary Table 5.a) and imatinib study (Supplementary Table 5.b).

To facilitate the correlation analysis, we generated scatter plots of reported percentages of top AEs in FAERS vs. ML model predicted probabilities of these AEs. The Spearman ρ was used to quantify the correlation by accommodating the potential nonlinear relationships (Supplementary Fig. 4). The results for all three studies indicate a clear correlation pattern (not purely linear) with significant correlations ($p < 0.05$).

Overall, the majority of top FAERS-reported AEs were captured by ML model with predicted high probability. Notably, for AEs specific to pharmacovigilance or AE with preferred terms not reported in clinical trials of the 16 SMKIs used for training the ML model, a predictive ML model cannot be built, and therefore no prediction can be made for these AEs.

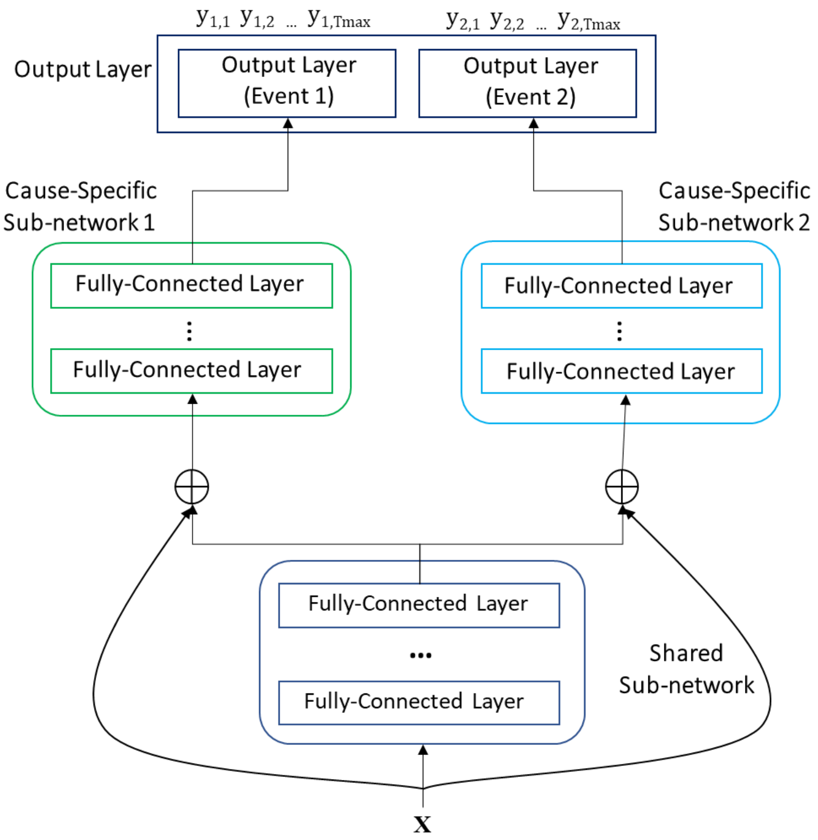

Supplementary Fig. 1 Architecture of a DeepHit model with two events for prediction. Adapted from [3] Copyright c 2018, Association for the Advancement of Artificial Intelligence.

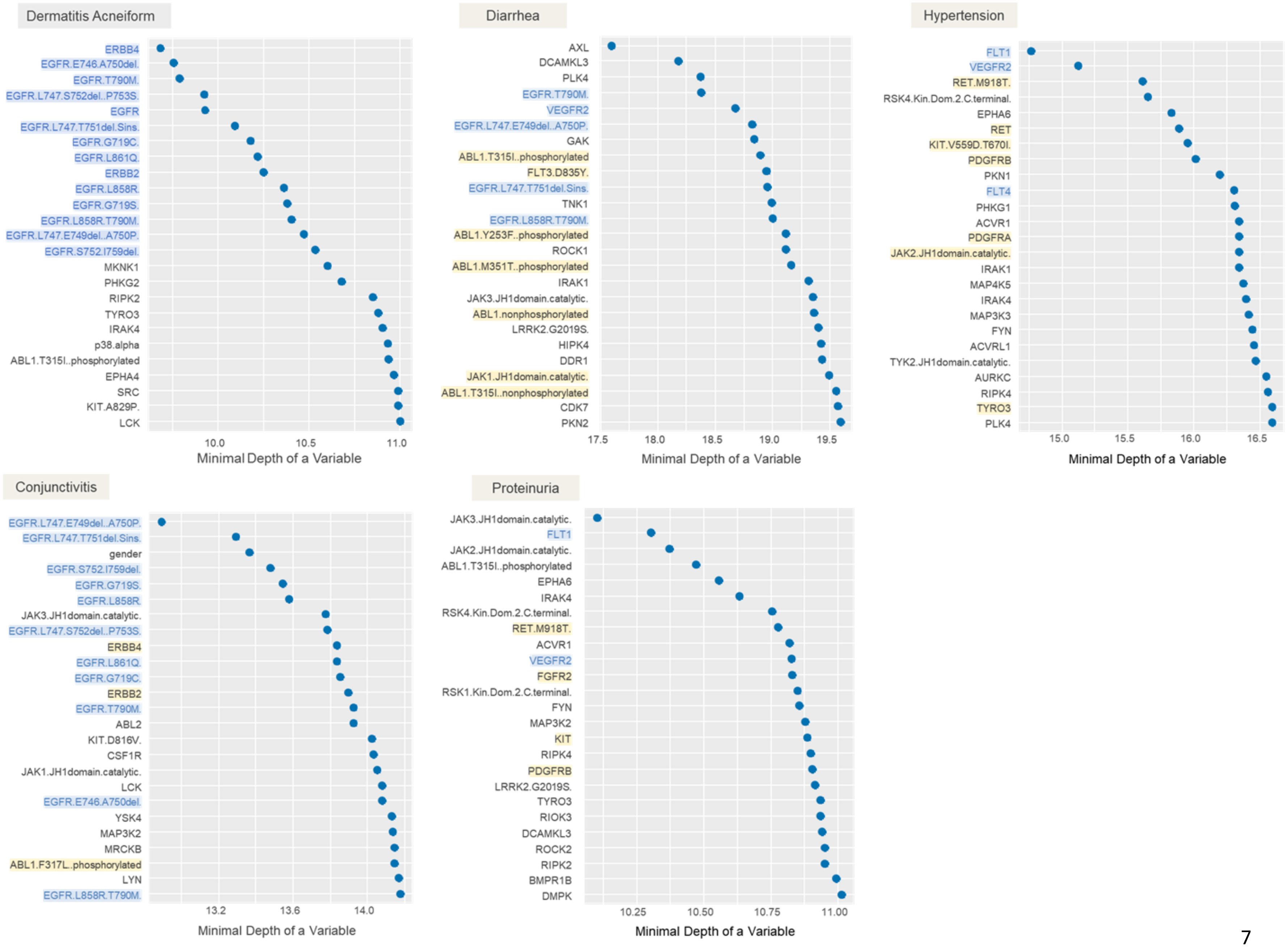

Dermatitis Acneiform
ERBB4
EGFR.E746.A750del.
EGFR.T790M.
EGFR.L747.S752del..P753S.
EGFR
EGFR.L747.T751del.Sins.
EGFR.G719C.
EGFR.L861Q.
ERBB2
EGFR.L858R.
EGFR.G719S.
EGFR.L858R.T790M.
EGFR.L747.E749del..A750P.
EGFR.S752.I759del.
MKNK1
PHKG2
RIPK2
TYRO3
IRAK4
p38.alpha
ABL1.T315I..phosphorylated
EPHA4
SRC
KIT.A829P.
LCK
10.0
10.5
11.0
Minimal Depth of a Variable
Diarrhea
AXL
DCAMKL3
PLK4
EGFR.T790M.
VEGFR2
EGFR.L747.E749del..A750P.
GAK
ABL1.T315I..phosphorylated
FLT3.D835Y.
EGFR.L747.T751del.Sins.
TNK1
EGFR.L858R.T790M.
ABL1.Y253F..phosphorylated
ROCK1
ABL1.M351T..phosphorylated
IRAK1
JAK3.JH1domain.catalytic.
ABL1.nonphosphorylated
LRRK2.G2019S.
HIPK4
DDR1
JAK1.JH1domain.catalytic.
ABL1.T315I..nonphosphorylated
CDK7
PKN2
17.5
18.0
18.5
19.0
19.5
Minimal Depth of a Variable
Hypertension
FLT1
VEGFR2
RET.M918T.
RSK4.Kin.Dom.2.C.terminal.
EPHA6
RET
KIT.V559D.T670I.
PDGFRB
PKN1
FLT4
PHKG1
ACVR1
PDGFRA
JAK2.JH1domain.catalytic.
IRAK1
MAP4K5
IRAK4
MAP3K3
FYN
ACVRL1
TYK2.JH1domain.catalytic.
AURKC
RIPK4
TYRO3
PLK4
15.0
15.5
16.0
16.5
Minimal Depth of a Variable
Conjunctivitis
EGFR.L747.E749del..A750P.
EGFR.L747.T751del.Sins.
gender
EGFR.S752.I759del.
EGFR.G719S.
EGFR.L858R.
JAK3.JH1domain.catalytic.
EGFR.L747.S752del..P753S.
ERBB4
EGFR.L861Q.
EGFR.G719C.
ERBB2
EGFR.T790M.
ABL2
KIT.D816V.
CSF1R
JAK1.JH1domain.catalytic.
LCK
EGFR.E746.A750del.
YSK4
MAP3K2
MRCKB
ABL1.F317L..phosphorylated
LYN
EGFR.L858R.T790M.
13.2
13.6
14.0
Minimal Depth of a Variable
Proteinuria
JAK3.JH1domain.catalytic.
FLT1
JAK2.JH1domain.catalytic.
ABL1.T315I..phosphorylated
EPHA6
IRAK4
RSK4.Kin.Dom.2.C.terminal.
RET.M918T.
ACVR1
VEGFR2
FGFR2
RSK1.Kin.Dom.2.C.terminal.
FYN
MAP3K2
KIT
RIPK4
PDGFRB
LRRK2.G2019S.
TYRO3
RIOK3
DCAMKL3
ROCK2
RIPK2
BMPR1B
DMPK
10.25
10.50
10.75
11.00
Minimal Depth of a Variable

Supplementary Fig. 2 Minimal depth assessment of n=314 predictive variables of for five representative AEs. The x-axis indicates the minimal depth measurement of the variable, which assesses the predictive power of a variable using in-bag data based on the distance from the root node of a tree to the first split on that variable; for each variable, the values are averaged over all trees in a forest. Smaller minimal depth indicates a variable has stronger impact on prediction accuracy. Top 25 variables were listed for each representative AE. The blue highlighted kinases are the representative experimentally well-established associations between kinase targets and AEs [7]; the yellow-highlighted pairs are validated by literature survey results for KI-AE pairs post the [7] publication (see Supplementary Table 3 for references). The results via minimal depth assessment are similar with the ones identified by VIMP (variable importance, in main text **Fig. 2**).

a

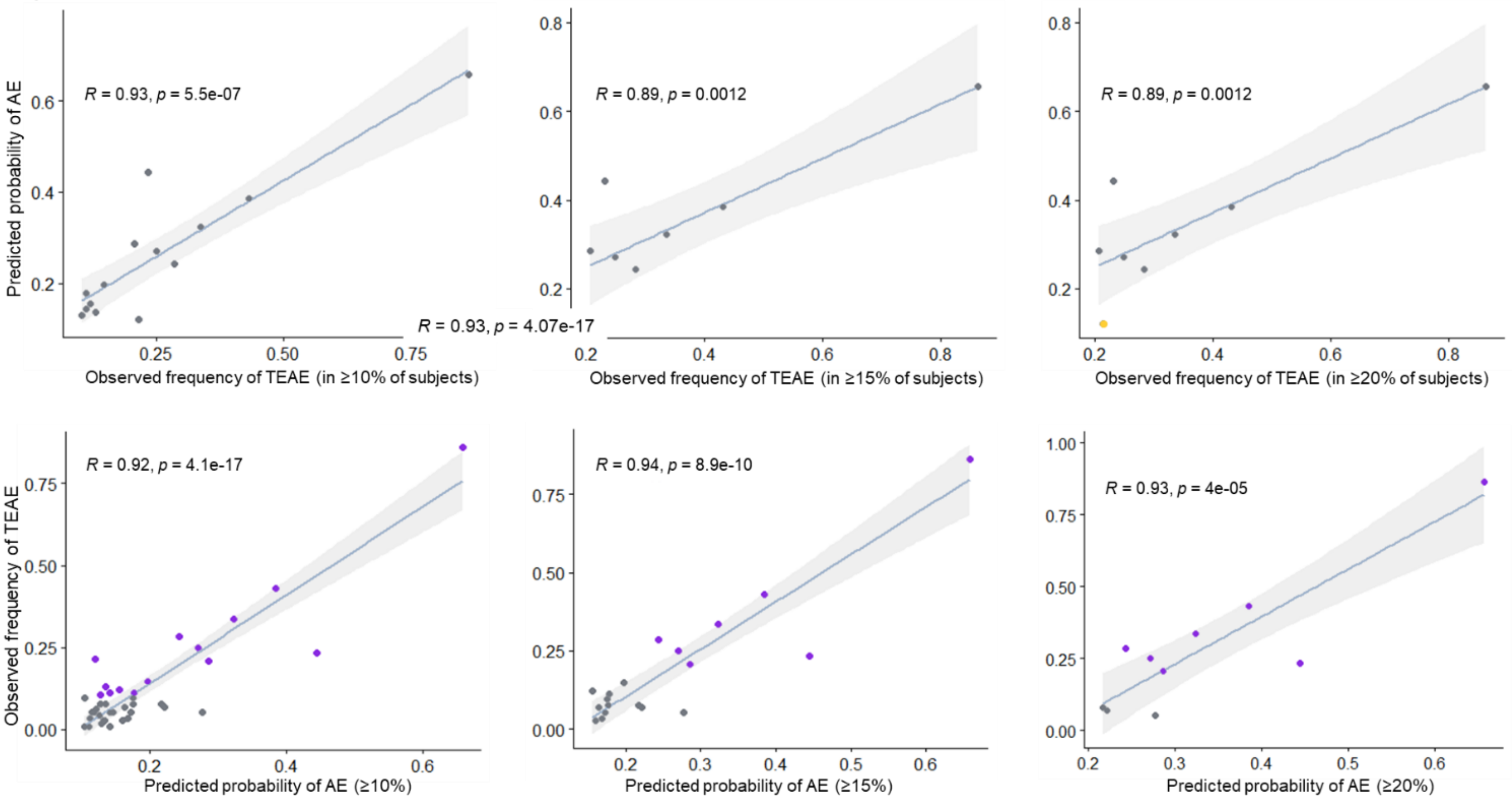
R = 0.93, p = 5.5e-07
Predicted probability of AE
Observed frequency of TEAE (in ≥10% of subjects)
R = 0.93, p = 4.07e-17
R = 0.89, p = 0.0012
Observed frequency of TEAE (in ≥15% of subjects)
R = 0.89, p = 0.0012
Observed frequency of TEAE (in ≥20% of subjects)
R = 0.92, p = 4.1e-17
Observed frequency of TEAE
Predicted probability of AE (≥10%)
R = 0.94, p = 8.9e-10
Predicted probability of AE (≥15%)
R = 0.93, p = 4e-05
Predicted probability of AE (≥20%)

b

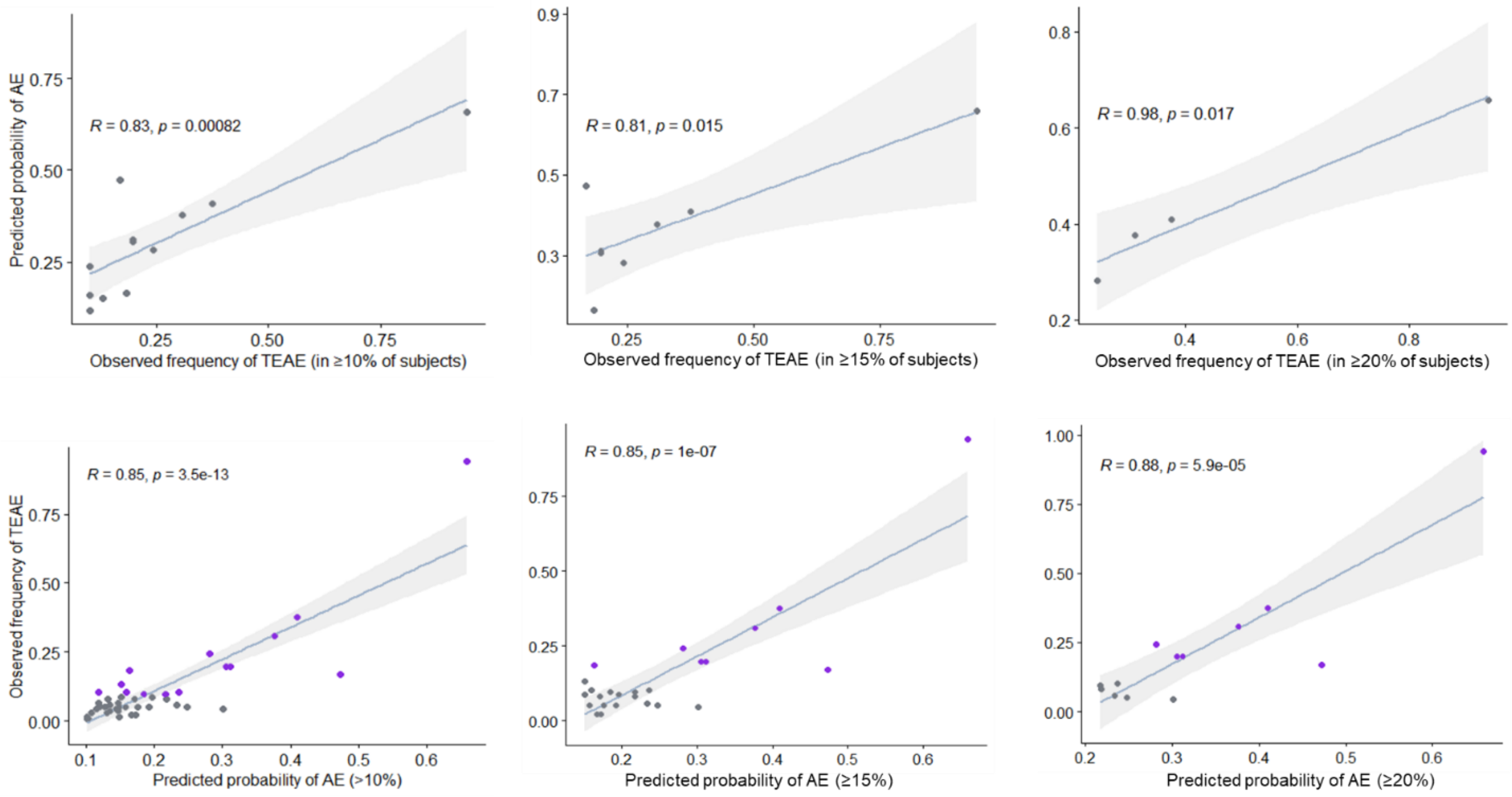
Predicted probability of AE
R = 0.83, p = 0.00082
Observed frequency of TEAE (in ≥10% of subjects)
R = 0.81, p = 0.015
Observed frequency of TEAE (in ≥15% of subjects)
R = 0.98, p = 0.017
Observed frequency of TEAE (in ≥20% of subjects)
Observed frequency of TEAE
R = 0.85, p = 3.5e-13
Predicted probability of AE (>10%)
R = 0.85, p = 1e-07
Predicted probability of AE (≥15%)
R = 0.88, p = 5.9e-05
Predicted probability of AE (≥20%)

c

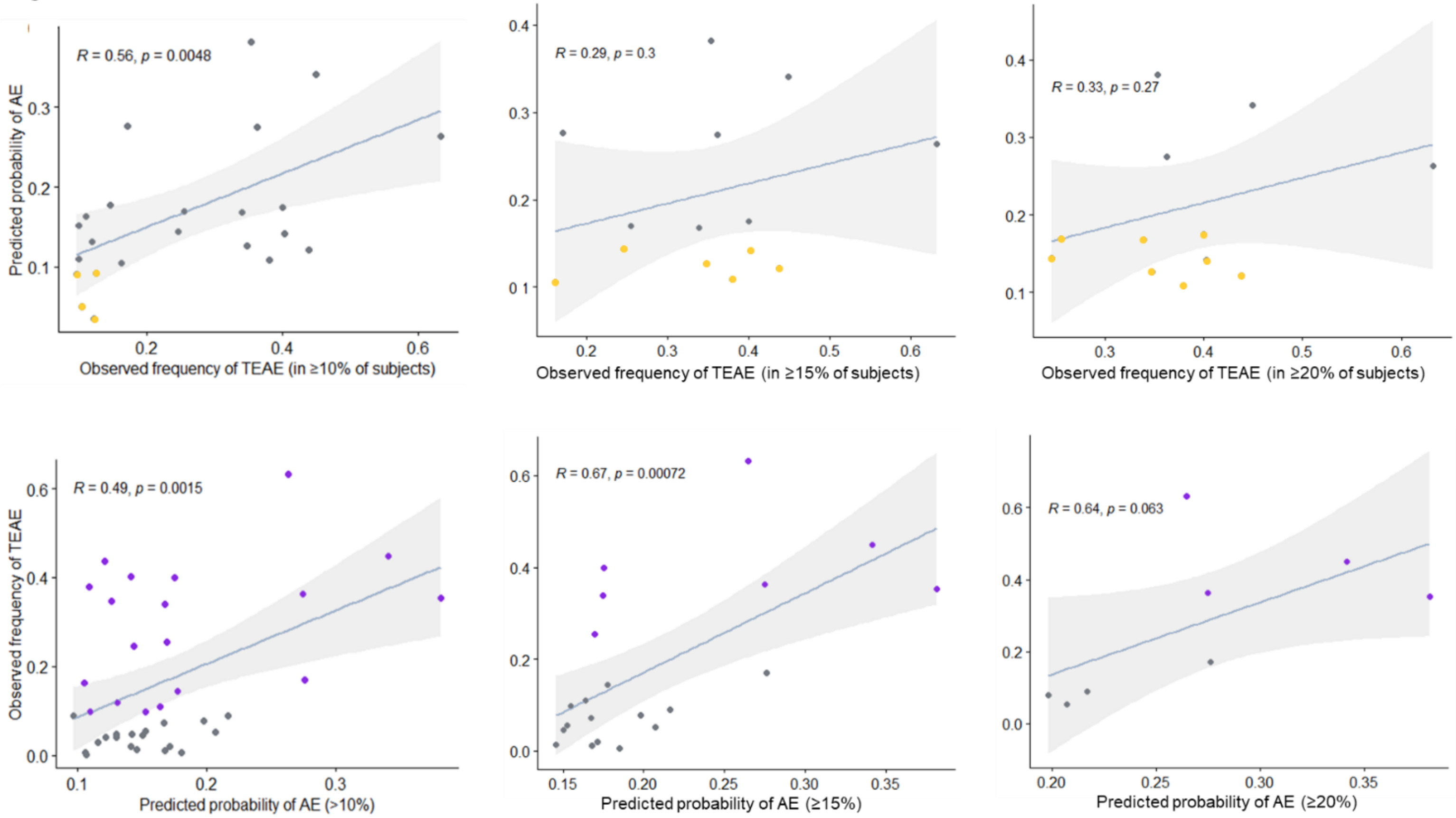

Supplementary Fig. 3 External validation on independent SMKI datasets: relationships between the model predicted AE probabilities and the observed AE incidences. Panels **a**, **b** and **c** are results based on two neratinib and one imatinib studies, respectively, corresponding to data used for generating Figure 3a, b and c in the main text. In each panel, the top row shows the scatterplots for the corresponding predicted probabilities vs all the frequently reported TEAEs (in ≥10%, ≥15%, ≥20% of study subjects) and the bottom row figures for the corresponding observed TEAEs vs all AEs predicted to occur in ≥10%, ≥15%, ≥20% of study subjects. On each plot, the blue line represents the fitted linear regression line, and the grey band represents the 95% confidence interval; Pearson's r (two-tailed) with p-value is displayed. Yellow points represent frequently reported TEAEs with predicted probabilities below the selected threshold values (i.e., 10%, 15% or 20%); purple points AEs with both predicted probabilities and observed incidences equal or above the threshold values (i.e., 10%, 15%, or 20%).

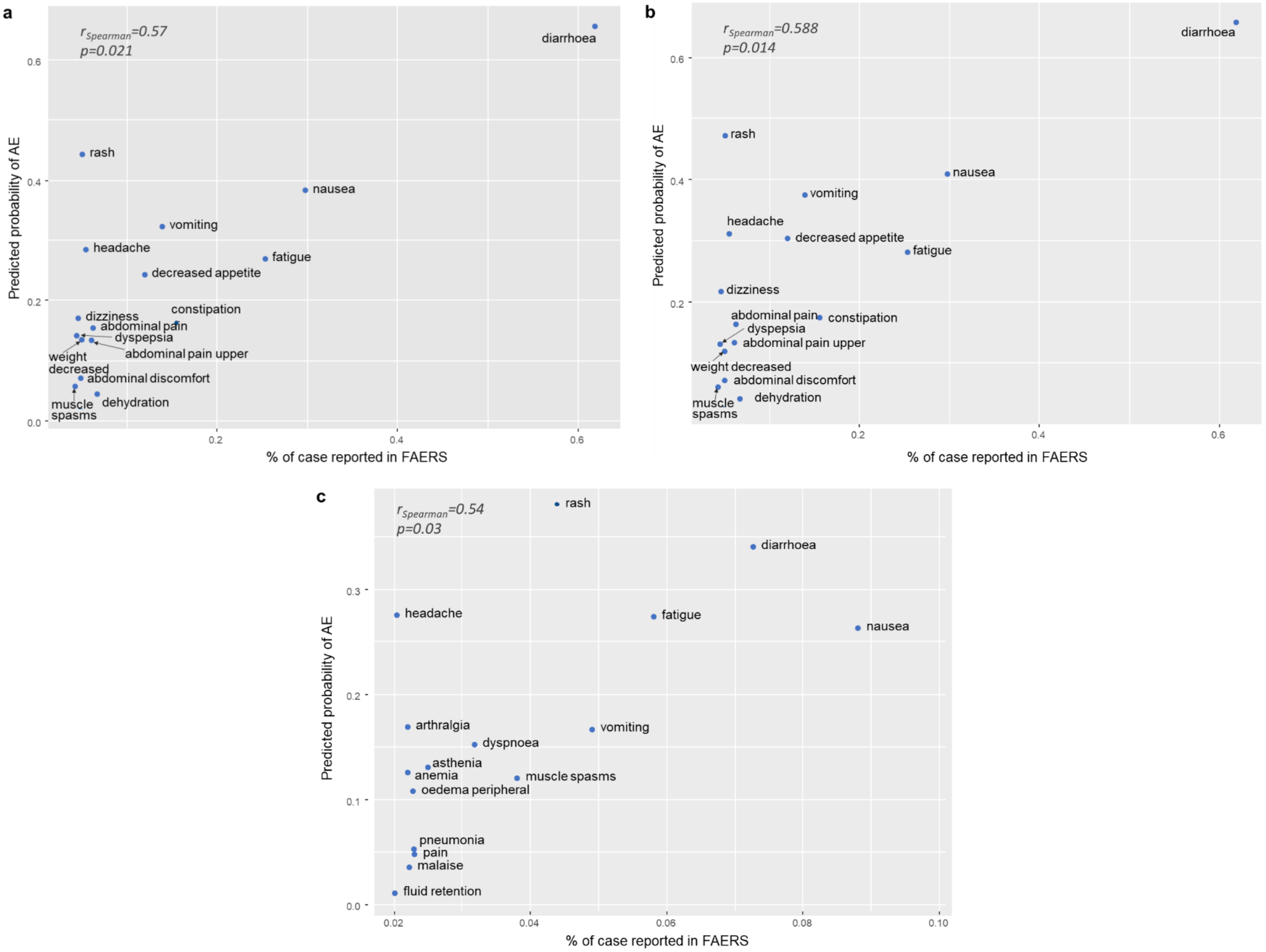
a
$r_{Spearman}$=0.57
p=0.021
diarrhoea
rash
nausea
vomiting
headache
fatigue
decreased appetite
constipation
dizziness
abdominal pain
dyspepsia
abdominal pain upper
weight decreased
abdominal discomfort
muscle spasms
dehydration
Predicted probability of AE
% of case reported in FAERS
b
$r_{Spearman}$=0.588
p=0.014
diarrhoea
rash
nausea
vomiting
headache
decreased appetite
fatigue
dizziness
abdominal pain
constipation
dyspepsia
abdominal pain upper
weight decreased
abdominal discomfort
muscle spasms
dehydration
Predicted probability of AE
% of case reported in FAERS
c
$r_{Spearman}$=0.54
p=0.03
rash
diarrhoea
headache
fatigue
nausea
arthralgia
vomiting
dyspnoea
asthenia
anemia
muscle spasms
oedema peripheral
pneumonia
pain
malaise
fluid retention
Predicted probability of AE
% of case reported in FAERS

Supplementary Fig. 4 Relationship between the model predicted AE probability and FAERS reported incidences. Sub-figures **a** and **b** show results for two neratinib based on n=16 AEs and **c** for one imatinib study based on n=16 AEs, corresponding to data used for generating Figure 3a, b and c in the main text. Spearman ρ (two-tailed) with p-value is displayed on each plot.

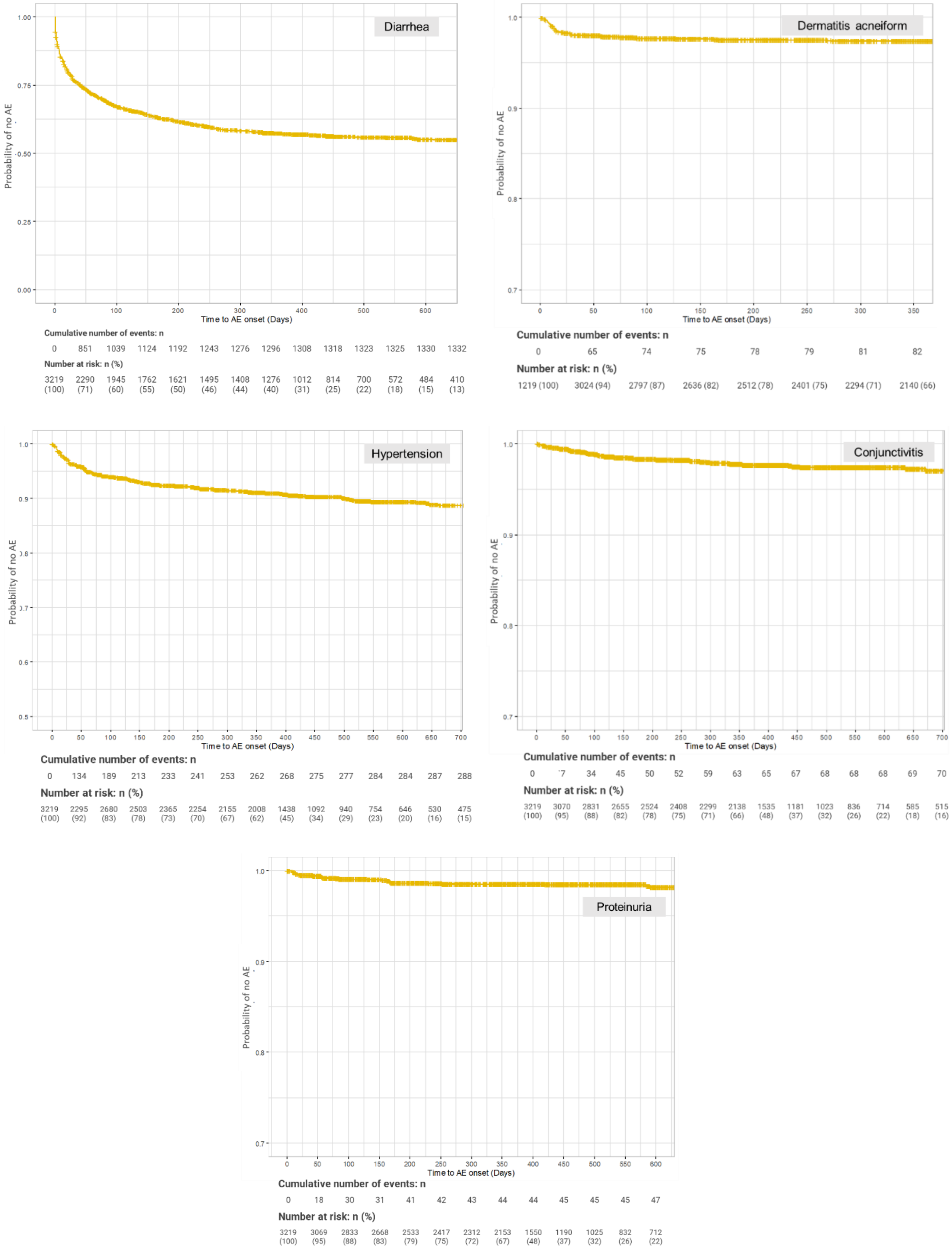

Supplementary Fig. 5 Kaplan–Meier survival curves for the patient population data for five representative AEs in Table 1.

Supplementary Table 1 List of SMKIs used for AE modeling

Drug Name	Primary Target*	Therapeutic Indication[a]	Year of First Approval	Approval procedure	Accessed Trial(s)[+]	*Kd* or %-inhibition
Axitinib (Inlyta)	VEGFR et al.	RCC	2012	Standard review	NCT00678392	Both
Cabozantinib (Cometriq, Cabometyx)	RET et al.	medullary thyroid cancer, RCC	2012	Priority review	NCT00704730	%-inhibition
Pazopanib (Votrient)	VEGFR	RCC, soft tissue sarcoma	2009	Standard review	NCT00334282	Both
Regorafenib (Stivarga)	VEGFR et al.	colorectal cancer, GIST	2012	Priority review Orphan drug	NCT01103323	%-inhibition
Sorafenib (Nexavar)	VEGFR et al.	RCC, hepatocellular carcinoma, thyroid cancer	2005	Priority review Orphan drug	NCT00984282	Both
Vandetanib (Caprelsa)	EGFR, VEGFR	medullary thyroid cancer	2011	Priority review Orphan drug	NCT00410761	Both
Afatinib (Gilotrif)	EGFR	NSCLC (EGFR L858R mutation–positive)	2013	Priority review Orphan drug	NCT00949650	Both
Crizotinib (Xalkori)	ALK	NSCLC (ALK–positive or ROS1-positive)	2011	Priority review Orphan drug	NCT00585195	Both

Erlotinib (Tarceva)	EGFR	NSCLC	2004	Rolling review	NCT00556712	Both
Dabrafenib (Tafinlar)	B-Raf	melanoma (BRAF V600E mutation-positive)	2013	Standard review<br>Orphan drug	NCT01227889	%-inhibition
Gefitinib (Iressa)	EGFR	NSCLC (EGFR L858R mutation–positive)	2003	Standard review<br>Orphan drug	NCT00206219	Both
Trametinib (Mekinist)	MEK	melanoma	2013	Standard review<br>Orphan drug	NCT01245062	%-inhibition
Vemurafenib (Zelboraf)	B-Raf	melanoma	2011	Priority review<br>Orphan drug	NCT01006980	%-inhibition
Bosutinib (Bosulif)	BCR-Abl	CML	2012	Standard review<br>Orphan drug	NCT00261846	Both
Dasatinib (Sprycel)	BCR-Abl et al.	CML	2006	Priority review<br>Orphan drug	NCT00481247	Both
Nilotinib (Tasigna)	BCR-Abl	CML	2007	Standard review<br>Orphan drug	NCT00471497	Both
Ruxolitinib (Jakafi)	JAK	myelofibrosis	2011	Priority review<br>Orphan drug	NCT01243944	Both
Nintedanib (Ofev)	EGFR et al.	idiopathic pulmonary fibrosis	2014	Priority review<br>Fast track & breakthrough therapy<br>Orphan drug	NCT01335464<br>NCT01335477	Kd

Midostaurin (Rydapt)	Flt3	acute myeloid leukemia, aggressive systemic mastocytosis	2017	Priority review<br>Fast track & breakthrough therapy<br>Orphan drug	NCT00782067<br>NCT03512197	Kd
Sunitinib (Sutent)	VEGFR et al.	RCC, GIST	2006	Priority review	NCT00077974	Both
Tofacitinib (Xeljanz)	JAK3	Rheumatoid arthritis	2012	Standard review	NCT00814307<br>NCT00856544<br>NCT00853385<br>NCT00847613<br>NCT00960440	Both

[*] The main targets of the SMKIs were determined using information in the drug labels as reference

[+] Studies are referred by their ClinicalTrials.gov Identifier.

[a] **RCC**: renal cell carcinoma **GIST**: gastrointestinal stromal tumor
NSCLC: Non-small cell lung cancer
CML: chronic myelogenous leukemia

[b] Source: IQVIA database

Supplementary Table 2 Predictive performances of the ML models on patient population data via C-index. The results are shown in mean of C-index (90% confidence interval). For each AE, the ML method with the best performance in terms of C-index is marked in bold.

AE (patients affected%)	DeepHit	ANN	RSF
Diarrhea (41.6)	0.651 (0.577, 0.724)	0.626 (0.524, 0.688)	**0.712** (0.689, 0.735)
Acne (2.4)	0.790 (0.743, 0.836)	**0.855** (0.781, 0.927)	0.852 (0.779, 0.912)
Hypertension (9.3)	**0.832** (0.822, 0.841)	0.792 (0.642, 0.853)	0.782 (0.751, 0.809)
Conjunctivitis (2.2)	**0.708** (0.607, 0.809)	0.685 (0.513, 0.815)	0.584 (0.510, 0.638)
Proteinuria (1.5)	0.815 (0.787, 0.843)	**0.816** (0.609, 0.912)	0.784 (0.696, 0.868)
Pneumonia (2.5)	0.568 (0.509, 0.626)	**0.594** (0.430, 0.741)	0.552 (0.445, 0.643)
Qt prolongation (2.5)	0.809 (0.770, 0.848)	0.793 (0.596, 0.891)	**0.836** (0.776, 0.887)
Headache (14.2)	0.576 (0.558, 0.595)	0.554 (0.443, 0.619)	**0.596** (0.554, 0.640)
Vomiting (17.0)	0.535 (0.420, 0.650)	0.694 (0.527, 0.748)	**0.739** (0.699, 0.776)
Fatigue (19.3)	**0.752** (0.740, 0.764)	0.697 (0.480, 0.756)	0.742 (0.711, 0.773)

Supplementary Table 3 Subset of the literature survey results with reported kinase-associated toxicities that match the ML model-identified KI-AE pairs (Figure 2 or Supplementary Fig. 2, yellow highlight)

Adverse event	Kinase	Reference
Dermatitis Acneiform	ErbB family	[8]
	ERBB4	[9]
Diarrhea	FLT3	[10-12]
	ERBB4	[9, 13]
	AXL	[12, 14]
	VEGFR2	[10, 14]
	BCR-ABL1	[15]
	JAK1, JAK2	[16]
Hypertension	FGFR	[10, 11, 17, 18]
	JAK1, JAK2, JAK3	[15, 16, 19]
	RET	[11, 12, 17]
	ABL1	[20, 21]
	TYRO3	[14]
	PDGFR, KIT	[11]
Conjunctivitis	ABL1	[22]
	ERBB2, ERBB4	[11]
Proteinuria	KIT, RET	[10, 23]
	FGFR1-4, PDGFR	[18]

Supplementary Table 4 Search parameters used in U.S. FAERS queries for the safety profile regarding the two SMKIs neratinib and imatinib.

Drug product	**neratinib**	**imatinib**
**NDA (new drug application) #**	N208051	N021588
**Approval Date**	Jul 17, 2017	Apr 18, 2003
**ANDA (abbreviated new drug application)**	None approved	Multiple (listed in the sub-table (a))
**Applicant Holder Full Name**	Puma Biotechnology Inc	Novartis Pharmaceuticals Corp
**Initial FDA Received Date**	07/17/2017 - 12/08/2021	04/18/2003 - 12/08/2021
**MedDRA Version**	24.1	24.1

(a) Approved ANDAs referencing to NDA #021588 listed in Orange Book [24]

**Market Status**	**Application No.**	**Product No.**	**Dosage Form**	**Route**	**Strength**	**TE Code**	**Applicant Holder**	**Approval Date**
RX	N021588	001	TABLET	ORAL	EQ 100MG BASE	AB	NOVARTIS PHARMACEUTICALS CORP	Apr 18, 2003
RX	N021588	002	TABLET	ORAL	EQ 400MG BASE	AB	NOVARTIS PHARMACEUTICALS CORP	Apr 18, 2003
RX	A079179	001	TABLET	ORAL	EQ 100MG BASE	AB	APOTEX INC	Aug 5, 2016
RX	A205990	001	TABLET	ORAL	EQ 100MG BASE	AB	BRECKENRIDGE PHARMACEUTICAL INC	Feb 8, 2019
RX	A206547	001	TABLET	ORAL	EQ 100MG BASE	AB	DR REDDYS LABORATORIES LTD	Aug 13, 2018
RX	A212773	001	TABLET	ORAL	EQ 100MG BASE	AB	EUGIA PHARMA SPECIALITIES LTD	Jul 23, 2020
RX	A207586	001	TABLET	ORAL	EQ 100MG BASE	AB	HIKMA PHARMACEUTICALS USA INC	Jul 13, 2018
RX	A204644	001	TABLET	ORAL	EQ 100MG BASE	AB	MYLAN PHARMACEUTICALS INC	Jun 21, 2017
RX	A207818	001	TABLET	ORAL	EQ 100MG BASE	AB	NATCO PHARMA LTD	Mar 1, 2019
RX	A208302	001	TABLET	ORAL	EQ 100MG BASE	AB	SHILPA MEDICARE LTD	Jan 17, 2019
RX	A078340	001	TABLET	ORAL	EQ 100MG BASE	AB	SUN PHARMACEUTICAL INDUSTRIES LTD	Dec 3, 2015
RX	A204285	001	TABLET	ORAL	EQ 100MG BASE	AB	TEVA PHARMACEUTICALS USA	Aug 4, 2016
RX	A208429	001	TABLET	ORAL	EQ 100MG BASE	AB	WOCKHARDT BIO AG	Jan 17, 2019
RX	A210658	001	TABLET	ORAL	EQ 100MG BASE	AB	ZYDUS PHARMACEUTICALS USA INC	Apr 8, 2020
RX	A079179	002	TABLET	ORAL	EQ 400MG BASE	AB	APOTEX INC	Aug 5, 2016
RX	A205990	002	TABLET	ORAL	EQ 400MG BASE	AB	BRECKENRIDGE PHARMACEUTICAL INC	Feb 8, 2019
RX	A206547	002	TABLET	ORAL	EQ 400MG BASE	AB	DR REDDYS LABORATORIES LTD	Aug 13, 2018

RX	A212773	002	TABLET	ORAL	EQ 400MG BASE	AB	EUGIA PHARMA SPECIALITIES LTD	Jul 23, 2020
RX	A207586	002	TABLET	ORAL	EQ 400MG BASE	AB	HIKMA PHARMACEUTICALS USA INC	Jul 13, 2018
RX	A204644	002	TABLET	ORAL	EQ 400MG BASE	AB	MYLAN PHARMACEUTICALS INC	Jun 21, 2017
RX	A207818	002	TABLET	ORAL	EQ 400MG BASE	AB	NATCO PHARMA LTD	Mar 1, 2019
RX	A208302	002	TABLET	ORAL	EQ 400MG BASE	AB	SHILPA MEDICARE LTD	Jan 17, 2019
RX	A078340	002	TABLET	ORAL	EQ 400MG BASE	AB	SUN PHARMACEUTICAL INDUSTRIES LTD	Dec 3, 2015
RX	A204285	002	TABLET	ORAL	EQ 400MG BASE	AB	TEVA PHARMACEUTICALS USA	Aug 4, 2016
RX	A208429	002	TABLET	ORAL	EQ 400MG BASE	AB	WOCKHARDT BIO AG	Jan 17, 2019
RX	A210658	002	TABLET	ORAL	EQ 400MG BASE	AB	ZYDUS PHARMACEUTICALS USA INC	Apr 8, 2020
DISCN	A207495	001	TABLET	ORAL	EQ 100MG BASE		AMNEAL PHARMACEUTICALS LLC	Feb 8, 2019
DISCN	A207495	002	TABLET	ORAL	EQ 400MG BASE		AMNEAL PHARMACEUTICALS LLC	Feb 8, 2019

Supplementary Table 5 Relationships between the model predicted AEs with high probability (≥10%) and the top reported AEs in U.S. FAERS. Sub-tables (a) and (b) are results based on two neratinib studies and one imatinib study, respectively.

(a) Neratinib studies

**Appear in model predicted AE with high probability (≥10%)**		**Preferred Terms**	**Total Cases reported in U.S. FAERS**	**% of Cases reported in U.S. FAERS**
**Study I**	**Study II**			
Yes		Diarrhea	824	61.80%
		Nausea	396	29.70%
		Fatigue	336	25.20%
		Constipation	206	15.50%
		Vomiting	184	13.80%
		Decreased appetite	158	11.90%
Not reported in the clinical trial*		Off label use	155	11.60%
		Death	128	9.60%
		Product dose omission issue	104	7.80%
5%	4%	Dehydration	88	6.60%
Yes		Abdominal pain	82	6.20%
		Abdominal pain upper	80	6.00%
		Headache	72	5.40%
Not reported in the clinical trial*		Hospitalisation	71	5.30%
Yes		Rash	66	5.00%
		Weight decreased	65	4.90%
7%	9%	Abdominal discomfort	64	4.80%
Yes		Dizziness	61	4.60%
		Dyspepsia	58	4.40%
6%	6%	Muscle spasms	56	4.20%

(b) Imatinib study

Appear in model predicted AE with high probability (≥10%)	Preferred Terms	Total Cases reported in U.S. FAERS	% of Cases reported in U.S. FAERS
Not reported in the clinical trial*	Death	2,069	9.7%
Yes	Nausea	1,875	8.8%
Yes	Diarrhea	1,546	7.3%
Yes	Fatigue	1,235	5.8%
Not reported in the clinical trial*	Drug ineffective	1,093	5.1%
Yes	Vomiting	1,043	4.9%
Yes	Rash	934	4.4%
Yes	Muscle spasms	809	3.8%
Yes	Dyspnoea	674	3.2%
Not reported in the clinical trial*	Neoplasm malignant	621	2.9%
Yes	Asthenia	530	2.5%
Yes	Pain	488	2.3%
Not reported in the clinical trial*	Illness	485	2.3%
5%	Pneumonia	485	2.3%
Yes	Oedema peripheral	483	2.3%
4%	Malaise	470	2.2%
Yes	Anaemia	467	2.2%
Yes	Arthralgia	466	2.2%
Yes	Headache	433	2.0%
1%	Fluid retention	427	2.0%

* The preferred terms were not reported in the clinical trials of the 16 SMKIs used for training the predictive ML model, therefore no predictive ML model were built, and no prediction can be made for these AE preferred terms.

+ The grey cells show top reported AEs in FAERS with model predicted probabilities of <0.1

Supplementary Table 6 Predictive performances of the machine learning model on patient population data assessed by C-index (using percent inhibition data, corresponding to **Table 1**)

AE (patients affected %)	Time to AE onset median [days] (range)	C-index (90% confidence interval)
Diarrhea (36.1)	25 (1, 616)	0.726 (0.701, 0.750)
Dermatitis acneiform (4.1)	14 (1, 634)	0.831 (0.769, 0.890)
Hypertension (14.2)	29 (1, 663)	0.799 (0.769, 0.826)
Conjunctivitis (2.2)	96 (3, 672)	0.637 (0.531, 0.741)
Proteinuria (2.2)	50 (1, 591)	0.853 (0.795, 0.904)

Additional Supplementary File

Supplementary Dataset 1: FAERS query result using search parameters in Supplementary Table 4. Tabs “PT_imatinib” and “PT_neratinib” display the case count and its percentage by preferred terms for imatinib and neratinib.

Supplementary References

1. Gong, X., M. Hu, and L. Zhao, *Big Data Toolsets to Pharmacometrics: Application of Machine Learning for Time-to-Event Analysis.* Clin Transl Sci, 2018. **11**(3): p. 305-311.
2. Lee, C., J. Yoon, and M.V. Schaar, *Dynamic-DeepHit: A Deep Learning Approach for Dynamic Survival Analysis With Competing Risks Based on Longitudinal Data.* IEEE Trans Biomed Eng, 2020. **67**(1): p. 122-133.
3. Lee, C., et al. *DeepHit: A Deep Learning Approach to Survival Analysis With Competing Risks*. in *AAAI Conference on Artificial Intelligence*. 2018.
4. Biganzoli, E., et al., *Feed forward neural networks for the analysis of censored survival data: a partial logistic regression approach.* Statistics in Medicine, 1998. **17**(10): p. 1169-1186.
5. Faraggi, D. and R. Simon, *A neural network model for survival data.* Stat Med, 1995. **14**(1): p. 73-82.
6. Chi, C.L., W.N. Street, and W.H. Wolberg. *Application of artificial neural network-based survival analysis on two breast cancer datasets.* in *AMIA Annu Symp*. 2007.
7. Dy, G.K. and A.A. Adjei, *Understanding, recognizing, and managing toxicities of targeted anticancer therapies.* CA Cancer J Clin, 2013. **63**(4): p. 249-79.
8. Arrieta, O., et al., *Randomized, open-label trial evaluating the preventive effect of tetracycline on afatinib induced-skin toxicities in non-small cell lung cancer patients.* Lung Cancer, 2015. **88**(3): p. 282-8.
9. Ran, F., et al., *Review of the development of BTK inhibitors in overcoming the clinical limitations of ibrutinib.* Eur J Med Chem, 2022. **229**: p. 114009.
10. Cabanillas, M.E., M. Ryder, and C. Jimenez, *Targeted Therapy for Advanced Thyroid Cancer: Kinase Inhibitors and Beyond.* Endocr Rev, 2019. **40**(6): p. 1573-1604.
11. Vergoulidou, M., *More than a Decade of Tyrosine Kinase Inhibitors in the Treatment of Solid Tumors: What We Have Learned and What the Future Holds.* Biomark Insights, 2015. **10**(Suppl 3): p. 33-40.
12. Weitzman, S.P. and M.E. Cabanillas, *The treatment landscape in thyroid cancer: a focus on cabozantinib.* Cancer Manag Res, 2015. **7**: p. 265-78.
13. Losanno, T. and C. Gridelli, *Safety profiles of first-line therapies for metastatic non-squamous non-small-cell lung cancer.* Expert Opin Drug Saf, 2016. **15**(6): p. 837-51.
14. Schwartz, G., et al., *Management of Adverse Events Associated with Cabozantinib Treatment in Patients with Advanced Hepatocellular Carcinoma.* Target Oncol, 2020. **15**(4): p. 549-565.
15. Gharwan, H. and H. Groninger, *Kinase inhibitors and monoclonal antibodies in oncology: clinical implications.* Nat Rev Clin Oncol, 2016. **13**(4): p. 209-27.
16. Hu, X., et al., *The JAK/STAT signaling pathway: from bench to clinic.* Signal Transduct Target Ther, 2021. **6**(1): p. 402.
17. Rutkowski, P. and J. Stepniak, *The safety of regorafenib for the treatment of gastrointestinal stromal tumors.* Expert Opin Drug Saf, 2016. **15**(1): p. 105-16.
18. Si, X., et al., *Management of anlotinib-related adverse events in patients with advanced non-small cell lung cancer: Experiences in ALTER-0303.* Thorac Cancer, 2019. **10**(3): p. 551-556.
19. Roger, I., et al., *The Role of JAK/STAT Molecular Pathway in Vascular Remodeling Associated with Pulmonary Hypertension.* Int J Mol Sci, 2021. **22**(9).
20. Eskazan, A.E., *Tyrosine kinase inhibitors (TKIs) used in the management of chronic myeloid leukaemia are associated with haematologic toxicities-Which TKI is the safest?* Br J Clin Pharmacol, 2019. **85**(10): p. 2241-2243.
21. Yuzbasioglu, M.B. and A.E. Eskazan, *Bosutinib - related pleural effusion in patients with chronic myeloid leukemia.* Expert Opin Drug Saf, 2021. **20**(4): p. 379-381.

22. Khoury, H.J., et al., *Cross-Intolerance With Dasatinib Among Imatinib-Intolerant Patients With Chronic Phase Chronic Myeloid Leukemia.* Clin Lymphoma Myeloma Leuk, 2016. **16**(6): p. 341-349 e1.
23. Takahashi, S., N. Kiyota, and M. Tahara, *Optimal use of lenvatinib in the treatment of advanced thyroid cancer.* Cancers Head Neck, 2017. **2**: p. 7.
24. Approved Drug Products with Therapeutic Equivalence Evaluations (Orange Book). https://www.fda.gov/Drugs/InformationOnDrugs/ucm129662.htm. Accessed Dec 2021
