# Supplementary material for: Decoding kinase-adverse event associations for small molecule kinase inhibitors
Source: Nat Commun. 2022 Jul 27;13:4349. doi: 10.1038/s41467-022-32033-5 (PMC9329312; doi:10.1038/s41467-022-32033-5)
Supplement: Supplementary file 2 — Reporting Summary [file 41467_2022_32033_MOESM2_ESM.pdf]

nature portfolio

Corresponding author(s): Liang Zhao

Last updated by author(s): Jul 1, 2022

Reporting Summary

Nature Portfolio wishes to improve the reproducibility of the work that we publish. This form provides structure for consistency and transparency in reporting. For further information on Nature Portfolio policies, see our Editorial Policies and the Editorial Policy Checklist.

Statistics

For all statistical analyses, confirm that the following items are present in the figure legend, table legend, main text, or Methods section.

n/a	Confirmed	
☐	☒	The exact sample size (*n*) for each experimental group/condition, given as a discrete number and unit of measurement
☒	☐	A statement on whether measurements were taken from distinct samples or whether the same sample was measured repeatedly
☐	☒	The statistical test(s) used AND whether they are one- or two-sided<br>*Only common tests should be described solely by name; describe more complex techniques in the Methods section.*
☒	☐	A description of all covariates tested
☒	☐	A description of any assumptions or corrections, such as tests of normality and adjustment for multiple comparisons
☐	☒	A full description of the statistical parameters including central tendency (e.g. means) or other basic estimates (e.g. regression coefficient) AND variation (e.g. standard deviation) or associated estimates of uncertainty (e.g. confidence intervals)
☐	☒	For null hypothesis testing, the test statistic (e.g. *F*, *t*, *r*) with confidence intervals, effect sizes, degrees of freedom and *P* value noted<br>*Give P values as exact values whenever suitable.*
☒	☐	For Bayesian analysis, information on the choice of priors and Markov chain Monte Carlo settings
☒	☐	For hierarchical and complex designs, identification of the appropriate level for tests and full reporting of outcomes
☒	☐	Estimates of effect sizes (e.g. Cohen's *d*, Pearson's *r*), indicating how they were calculated

Our web collection on statistics for biologists contains articles on many of the points above.

Software and code

Policy information about availability of computer code

Data collection	FDA internal eCTD (electronic Common Technical Document) software was used to download individual-level data in the clinical trials.
Data analysis	Individual drug exposure were obtained using population PK modeling implemented in NONMEM (Version 7.4).<br>The severity of adverse events was graded using Common Terminology Criteria for Adverse Events (CTCAE, Version 3.0).<br>Data integration and analyses were performed using custom scripts and open source packages in R 3.6.0 and Python 3.7.9. R packages used include dplyr 1.0.7, survival 2.3-11, randomForestSRC 2.13.0, nnet 7.3, survminer 0.4.8, ggplot2 3.3.2, survcomp 1.44.0; Python libraries used include numpy 1.19.2, pandas 1.1.15, lifelines 0.26.4, sklearn 1.0.1, DeepHit (https://github.com/chl8856/DeepHit)

For manuscripts utilizing custom algorithms or software that are central to the research but not yet described in published literature, software must be made available to editors and reviewers. We strongly encourage code deposition in a community repository (e.g. GitHub). See the Nature Portfolio guidelines for submitting code & software for further information.

Data

Policy information about availability of data

All manuscripts must include a data availability statement. This statement should provide the following information, where applicable:

- Accession codes, unique identifiers, or web links for publicly available datasets
- A description of any restrictions on data availability
- For clinical datasets or third party data, please ensure that the statement adheres to our policy

The data used in this study were derived from the third-party registrational clinical trials data (including individual-level data), which cannot be made publicly available due to confidentiality obligations and regulation policies in FDA. The processed datasets may be available under restricted access for bona fide research. Specific requests for de-identified population-level data should be sent to the corresponding author upon ethical review. All requests will be promptly reviewed within 15 working days. We have made secondary data and information publicly accessible via the web application "Identification of Kinase-Specific Signal" (https://gongj.shinyapps.io/ml4ki).

Human research participants

Policy information about studies involving human research participants and Sex and Gender in Research.

Reporting on sex and gender	N/A
Population characteristics	N/A
Recruitment	N/A
Ethics oversight	N/A

Note that full information on the approval of the study protocol must also be provided in the manuscript.

Field-specific reporting

Please select the one below that is the best fit for your research. If you are not sure, read the appropriate sections before making your selection.

☒ Life sciences ☐ Behavioural & social sciences ☐ Ecological, evolutionary & environmental sciences

For a reference copy of the document with all sections, see nature.com/documents/nr-reporting-summary-flat.pdf

Life sciences study design

All studies must disclose on these points even when the disclosure is negative.

Sample size	Sample sizes were not pre-determined. Sample sizes were based on the availability of individual-level data in the registrational clinical trials included in the study.
Data exclusions	Within each clinical trial, the patients who had both safety data and at least one evaluable PK (pharmacokinetics) measurement available were included in the analysis, i.e., patients with either safety data or PK data missing were excluded.
Replication	Reproducible data integration, predictive model building and performance evaluation pipeline was automated using R scripts. The data and metrics reported in the manuscript have been independently reproduced by data analytics team in the corresponding author's affiliation.
Randomization	Randomization was not relevant to the study, as we used data previously collected data in the clinical trials and did not conduct any additional clinical trial.
Blinding	Blinding was not relevant to the study as we did not conduct any clinical trial.

Reporting for specific materials, systems and methods

We require information from authors about some types of materials, experimental systems and methods used in many studies. Here, indicate whether each material, system or method listed is relevant to your study. If you are not sure if a list item applies to your research, read the appropriate section before selecting a response.

Materials & experimental systems

n/a	Involved in the study
☒	☐ Antibodies
☒	☐ Eukaryotic cell lines
☒	☐ Palaeontology and archaeology
☒	☐ Animals and other organisms
☒	☐ Clinical data
☒	☐ Dual use research of concern

Methods

n/a	Involved in the study
☒	☐ ChIP-seq
☒	☐ Flow cytometry
☒	☐ MRI-based neuroimaging
