# Supplementary material for: Decoding kinase-adverse event associations for small molecule kinase inhibitors
Source: Nat Commun. 2022 Jul 27;13:4349. doi: 10.1038/s41467-022-32033-5 (PMC9329312; doi:10.1038/s41467-022-32033-5)
Supplement: Supplementary file 3 — Description of Additional Supplementary Files [file 41467_2022_32033_MOESM3_ESM.pdf]

Title: Supplementary Dataset 1

Description: FAERS Query Result Using Search Parameters in Supplementary Table 4
